# Self-enforcing HMGB1/NF-κB/HIF-1α Feedback Loop Promotes Cisplatin Resistance in Hepatocellular Carcinoma Cells

**DOI:** 10.7150/jca.42944

**Published:** 2020-04-06

**Authors:** Yang Song, Xuejing Zou, Dongyan Zhang, Shanshan Liu, Zhijiao Duan, Li Liu

**Affiliations:** 1 Hepatology Unit and Department of Infectious Diseases, Nanfang Hospital, Southern Medical University, Guangzhou 510515, China; 2 Department of Radiation Oncology, Nanfang Hospital, Southern Medical University, Guangzhou, PR China.

**Keywords:** HMGB1, Hepatocellular carcinoma, Cisplatin resistance, HIF-1α, NF-κB, Self-enforcing

## Abstract

Hepatocellular carcinoma (HCC) is ranked the sixth most common cancer and the fourth leading cause of cancer-related death worldwide, and its incidence is expected to increase in the future. Cisplatin has been widely used in chemotherapy and transarterial chemoembolization in treatment for HCC. However, the main obstacle to the clinical use of cisplatin is the development of resistance, the mechanisms of which are poorly defined. Therefore, it is imperative to investigate the cellular mechanisms mediating cisplatin resistance in HCC. Here, we demonstrated that high mobility group box 1 (HMGB1) is upregulated in patients with cancer, and implicated in a tumor-supportive role. Further, we showed that HMGB1 has an important role in mediating cisplatin resistance via an HMGB1/ nuclear factor kappa-B (NF-κB)/ hypoxia inducible factor-1α (HIF-1α) feedback loop. The study findings reveal an unappreciated molecular mechanism of HMGB1-mediated cisplatin resistance and may provide a new clue in cancer therapy.

## Introduction

Hepatocellular carcinoma (HCC) is the sixth most common cancer, and the fourth leading cause of cancer-related death worldwide, with more than 840,000 new cases diagnosed and 781,000 deaths in 2018 1. Although there is a wide range of available therapeutic options for HCC, chemotherapy is nonetheless still often adopted in patients with highly advanced HCC 2. Chemotherapeutic agents such as cisplatin are widely used in transarterial chemoembolization 3. However, chemotherapy often fails due to multidrug resistance (MDR), leading to a poor prognosis in patients with advanced disease 3, 4. Therefore, it is imperative to investigate the cellular mechanisms mediating cisplatin resistance in HCC.

High mobility group box 1 (HMGB1) is a nonhistone chromatin-binding protein that shows high electrophoretic mobility when run on polyacrylamide gels 5. HMGB1 has multiple functions depending on its location 6. In the nucleus, HMGB1 takes part in DNA replication, repair, recombination, transcription, and genomic stability 7, 8. Cytoplasmic HMGB1 expression has a role in autophagy regulation in cancer cells 9, 10. In addition, extracellular HMGB1, after either active release from cells or passive release upon lytic cell death, functions as a pro-inflammatory cytokine and perpetuates inflammatory responses in a wide variety of pathologies 11. Owing to its versatile role in cancer, HMGB1 has been proposed as a potential cancer biomarker of survival and a target for cancer therapy 12. Although the role of HMGB1 in tumorigenesis, progression, invasion, metastasis, and prognosis in different malignancies has been frequently discussed, its function in cisplatin resistance in HCC is not fully understood 13-15. Understanding the role of HMGB1 is necessary to address the current problem of cisplatin resistance.

In the present study, we found that HMGB1 expression was prominently induced in HCC cells treated with cisplatin and significantly upregulated in HCC tissues than in matched adjacent liver tissues. Functionally, HMGB1 silencing decreased cancer cell proliferation. HMGB1 markedly encouraged resistance to cisplatin by protecting HCC cells from apoptosis. Mechanistically, we report the identification of a positive HMGB1/NF-κB/HIF-1α feedback loop and define its critical role in cisplatin resistance.

## Materials and Methods

### Cell culture

The human hepatocarcinoma cell lines SK-Hep-1, SMMC-7721, and Huh7, and the hepatocyte cell line HL-7702 were purchased from the Cell Bank of Type Culture Collection (Shanghai, China). SK-Hep-1 and Huh7 cells were cultured in Dulbecco's modified eagle medium (DMEM, Gibco, MA USA); SMMC-7721 cells were incubated in RPMI 1640 (Thermo Fisher, MA USA), supplemented with 10% fetal bovine serum (FBS, Biological Industries, CT USA) in a moist incubator with 5% CO_2_.

### Western blot analysis

The extraction of HCC cell total proteins was acquired using RIPA lysis buffer (Cell Signaling Technology, MA USA) containing protease and phosphatase inhibitors (Roche, Basel, Switzerland). Protein concentration was determined by the BCA Protein Assays kit (ThermoFisher, MA USA). Lysates were separated on a 10% sodium dodecyl sulfate-polyacrylamide gel and electro-transferred to a polyvinylidene difluoride membrane (Bio-Rad, CA USA) that was blocked with 5% skimmed milk for 1 hour at room temperature and cultured overnight at 4°C with primary antibodies followed by incubation with horseradish peroxidase-conjugated secondary antibodies (Abcam, MA USA). Immunodetection was performed using chemiluminescence detection system (Bio-Rad, CA USA). Antibodies include HMGB1, (1:1000, from Proteintech, IL USA), PARP, cleaved-PARP (1:1000, from Abcam, MA USA), NF-κB-p65, phosphor-NF-κB-p65 (1:1000, from CST, MA USA), and HIF-1α (1:1000, from BD Biosciences, CA USA); β-actin was an internal control.

### Reagents

Cisplatin (Sigma, MA USA), stable in sterile solutions in 0.9% sodium chloride protected from light for 28 days at room temperature. TNF-α (PeproTech, NJ USA), dissolved in phosphate-buffered saline (PBS) to 10μg/μL stock at -20°C. Cobalt (II) chloride hexahydrate (Sigma, MA USA), dissolved in deionized water to 20mM, freshly prepared solution and used at room temperature.

### Clinical human tissue samples

Six pairs of tumor tissues and adjacent normal tissues were obtained from Nanfang Hospital, Southern Medical University, Guangzhou, China from June 2017 to July 2018, and the informed consent was signed by each participant. This experiment was permitted by the ethics committee of the Nanfang Hospital. Patients who met the following inclusion criteria were enrolled: (I) received liver resection for primary HCCs for the first time; (II) no evidence of extrahepatic metastasis at the initial stage; (III) Child- Pugh class A of liver function at the initial stage. Patients who received radiotherapy or chemotherapy before surgery were excluded. The resulting tissue samples were frozen in liquid nitrogen immediately.

### Cancer biostatistical analysis

Relative expression levels of HMGB1 in hepatocellular carcinoma patients compared to normal samples were analyzed using previously published microarray database (GEO: GSE25097, IGCG: IGCG-LIRI-JP). Patients were divided into low-HMGB1 and high-HMGB1 groups based on HMGB1 expression for overall survival analysis.

The linear regression was analyzed using data from the GEO: GSE364 dataset.

### Cell small interfering RNA transfection

Specific siRNAs against HMGB1 (si-HMGB1#1 and si-HMGB1#2) or HIF-1α (si-HIF-1α) and their corresponding NCs (si-NC) were acquired from RiboBio (Guangzhou, China). Cells were transfected with these siRNAs through Lipofectamine RNAiMAX (Invitrogen, MA USA) for 48h.

### Cell plasmid transfection

The HMGB1 and P65 overexpression plasmid was purchased from Genechem (Shanghai, China). The plasmid was used to transfect well-growing state cells by using Lipofectamine 3000 transfection reagent (Invitrogen, MA USA) for 48h.

### 5-ethynyl-20-deoxyuridine (EdU) assays

Transfected SK-Hep-1 or SMMC-7721 cells were incubated for 2 h by use of 50 μM EdU (RiboBio, Guangzhou China), followed by Apollo staining (RiboBio, Guangzhou China) and then staining with DAPI (ZSGB-Bio, Beijing China). At length, EdU-positive cells were revealed via a fluorescence microscope (Oympus, Tokyo Japan) in eight random fields. Four independent experiments were performed.

### CCK8 assay

The CCK8 assay was carried out following the manufacturer's instructions (Dojindo Laboratories, Osaka Japan). Absorbance of each well was quantified at 450 nm by an enzyme-labeled instrument, and cell growth was assayed every day for five days.

### Real-time polymerase chain reaction (RT-PCR)

Total RNA was extracted from cell lines and isolated by TRIzol reagent (Invitrogen, MA USA) according to the manufacturer's protocol. qRT-PCR was performed using the SYBR Green PCR kit (Takara Biotechnology, Tokyo Japan). β-actin was used as internal control for NF-κB and HIF-1α. The specificity of amplification products was confirmed by melting curve analysis. Triplicate samples were analyzed in three independent experiments.

### Statistical analysis

The quantitative data are presented as the means ± SD of at least three independent experiments. The statistical analyses were performed using SPSS V.19.0 software and GraphPad Prism 8. Student's t test was applied to compare the significant differences between two groups, and analysis of variance (ANOVA) was used for multiple comparisons. All tests were two-sided, and differences were considered statistically significant at a *p*-value below 0.05.

## Results

### HMGB1 is upregulated in cancer cells treated with cisplatin and human cancer tissues

**Figure 1:**
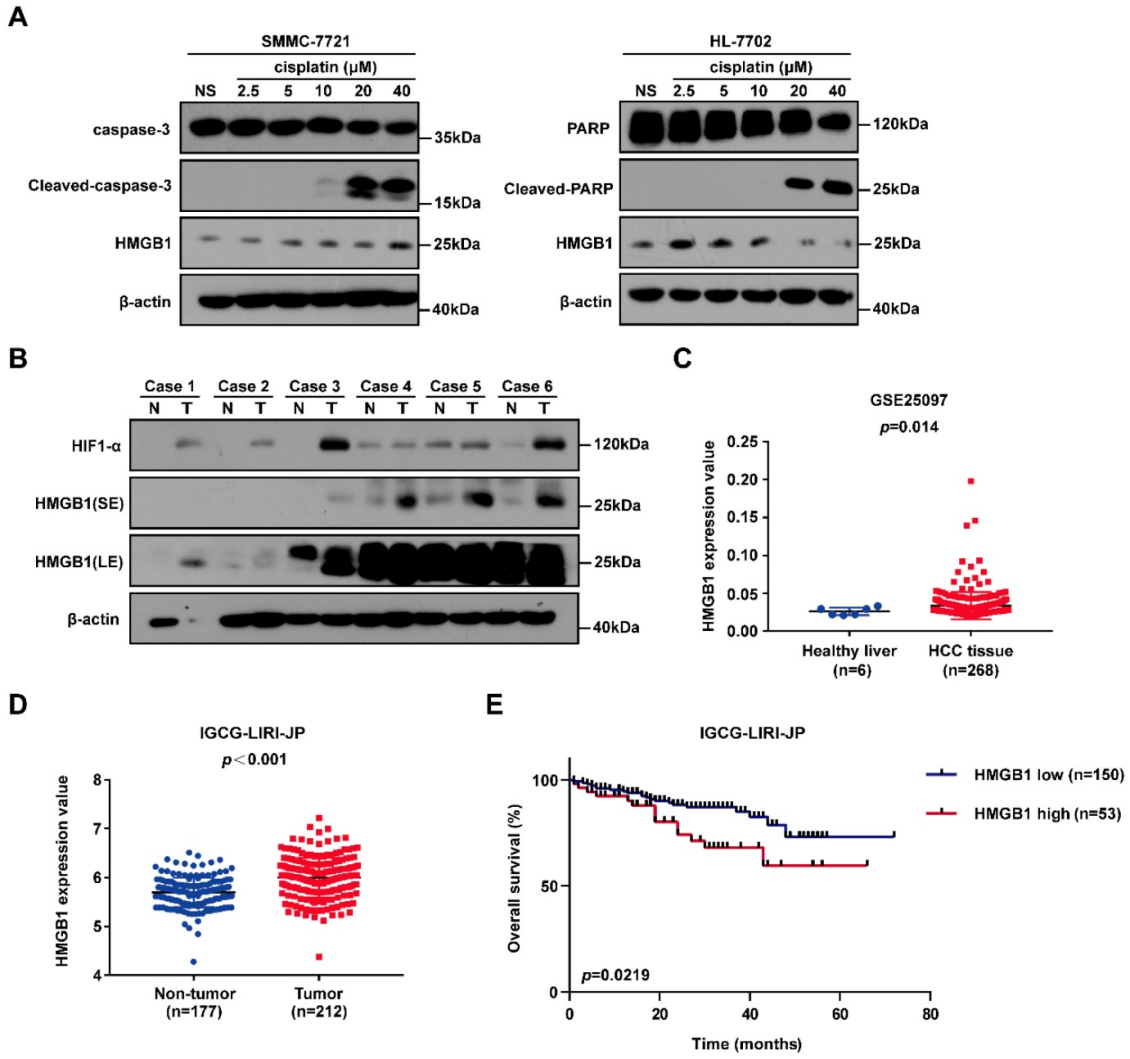
HMGB1 is upregulated in cancer cells treated with cisplatin and human cancer tissues. (A) Lysates from SMMC-7721 cells and HL-7702 cells treated with graduate increased dosage of cisplatin were subjected to immunoblotting. NS: normal saline. (B) the expression of HIF-1α and HMGB1 in 6 pairs of HCC tissues (T) and their matched normal-appearing tissues (N). SE: short exposure, LE: long exposure. (C) HMGB1 expression in healthy liver and HCC tissue using the GSE25097 (n=6 or 268) cohort. p Value was determined by unpaired two-sample Student's *t* test. (D) HMGB1 expression in non-tumor tissue (n=177) and tumor tissue (n=212) using the IGCG-LIRI-JP cohort. *p* Value was determined by Student's t test. (E) Analysis of overall survival (OS) in hepatocellular carcinoma patients with high or low HMGB1 expression using the IGCG-LIRI-JP (n=53 or 150) cohort. *p* Value was determined by log-rank (Mantel-Cox) test.

To identify the gene involved in cisplatin resistance of liver cancer cells, we first treated SMMC-7721 cancer cells and normal human HL-7702 hepatocytes with increasing doses of cisplatin. Immunoblotting showed that HMGB1 protein levels were significantly higher in SMMC-7721 cells than in HL-7702 cells, suggesting that HMGB1 may be related to cisplatin resistance (Fig. 1A). Next, we explored the expression of HMGB1 in tumors. Immunoblotting showed the upregulation of HMGB1 in HCC tumor samples of the patients. Moreover, HIF-1α upregulation was observed (Fig. 1B). For further verification, we analyzed data from the GSE25097 and IGCG-LIRI-JP datasets; both of them showed that HMGB1 expression was increased in HCC tissues (Figs. 1C and 1D). More importantly, to investigate the clinical importance of HMGB1, we performed a survival analysis based on data from the IGCG-LIRI-JP dataset. The survivorship curve showed that patients with high HMGB1 expression exhibited poorer overall survival (OS) (Fig. 1E). Multivariate analysis results showed that OS in the IGCG-LIRI-JP cohort could be predicted based on significant prognostic factors, such as portal vein invasion, alcohol consumption, and HMGB1 expression (Table 1 and Table 2). Taken together, these results suggested that HMGB1 may be related to cisplatin resistance and that in a substantial proportion of HCCs, HMGB1 is upregulated and associated with poor clinical outcomes.

### HMGB1 silencing decreases cancer cell proliferation

**Figure 2:**
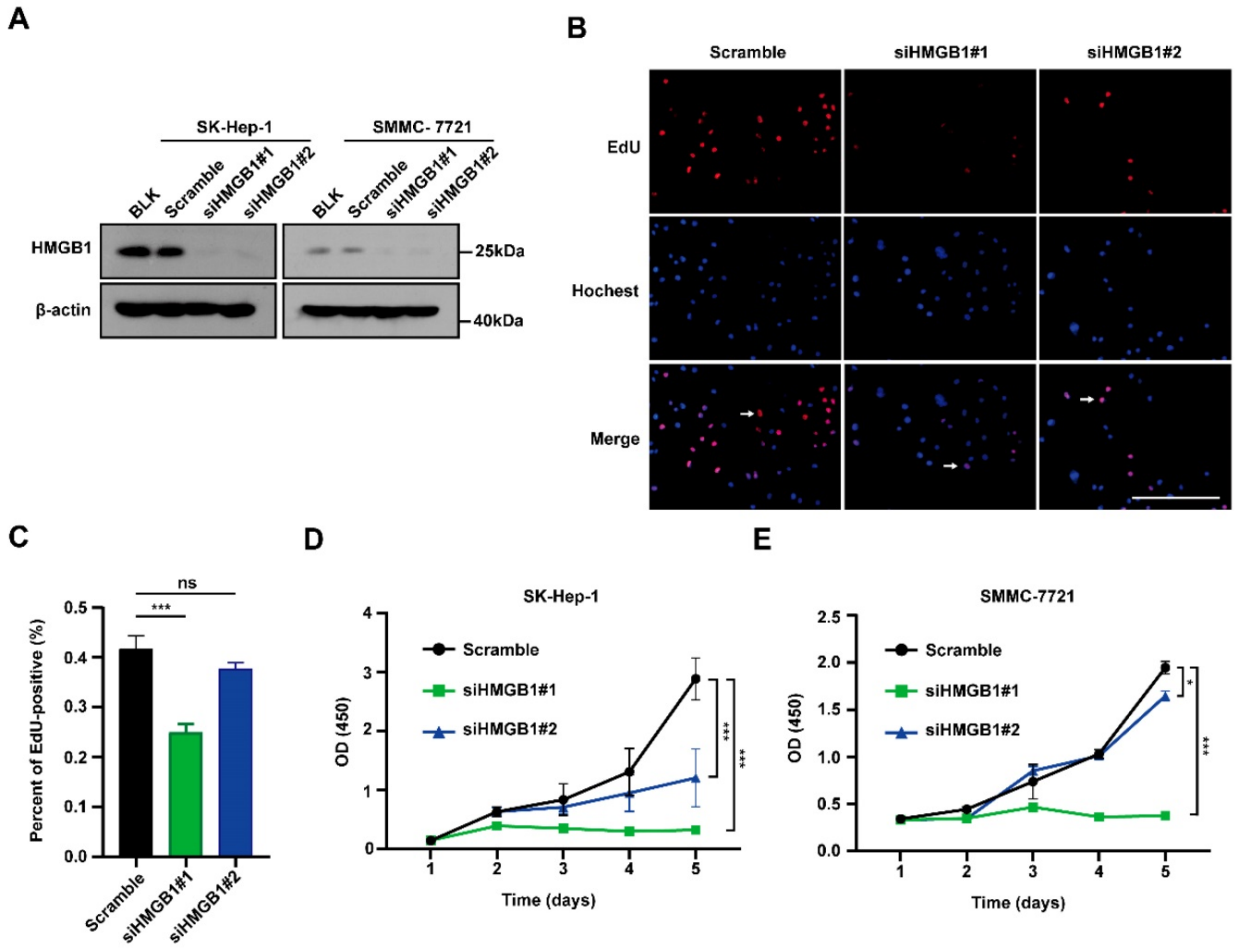
Silencing of HMGB1 decreases cancer cells proliferation. (A) Validation of two independent HMGB1 siRNAs by immunoblotting. (B) The percentage of S phase cell in HCC cells transfected with siRNAs against HMGB1 was detected by the EdU incorporation assays. Scale bars, 100μm. Arrows: EdU-positive cells. (C) Quantification of the number of EdU-positive cells is shown in the right. n=8, *p* Value was determined by Student's t test (*p < 0.05, ** p < 0.01, *** p < 0.001, ns: not significant). (D-E) The proliferation ability of with or without knockdown HMGB1 in SK-Hep-1 and SMMC-7721 cells evaluated by CCK8 assay at various time points. The experiments were performed by triplicate. *p* Value was determined by factorial design ANOVA. (*p < 0.05, ** p < 0.01, *** p < 0.001, ns: not significant).

To examine the biological functions of HMGB1 in tumors, first SMMC-7721 and SK-Hep-1 cells were transfected with HMGB1 siRNA; HMGB1 expression levels were successfully reduced in both systems by immunoblotting (Fig. 2A). We then conducted EdU incorporation assays to assess HCC cell proliferations. The percentage of EdU-positive cells was significantly reduced in the HMGB1-silenced group than in the control group (Figs. 2B and 2C). In addition, we obtained the same result using CCK8 assays in SK-Hep-1 and SMMC-7721 cells (Fig. 2D and 2E). The above results show that HMGB1 silencing decreased cancer cell proliferation.

### HMGB1 confers cisplatin resistance to HCC cells via HIF-1α

**Figure 3:**
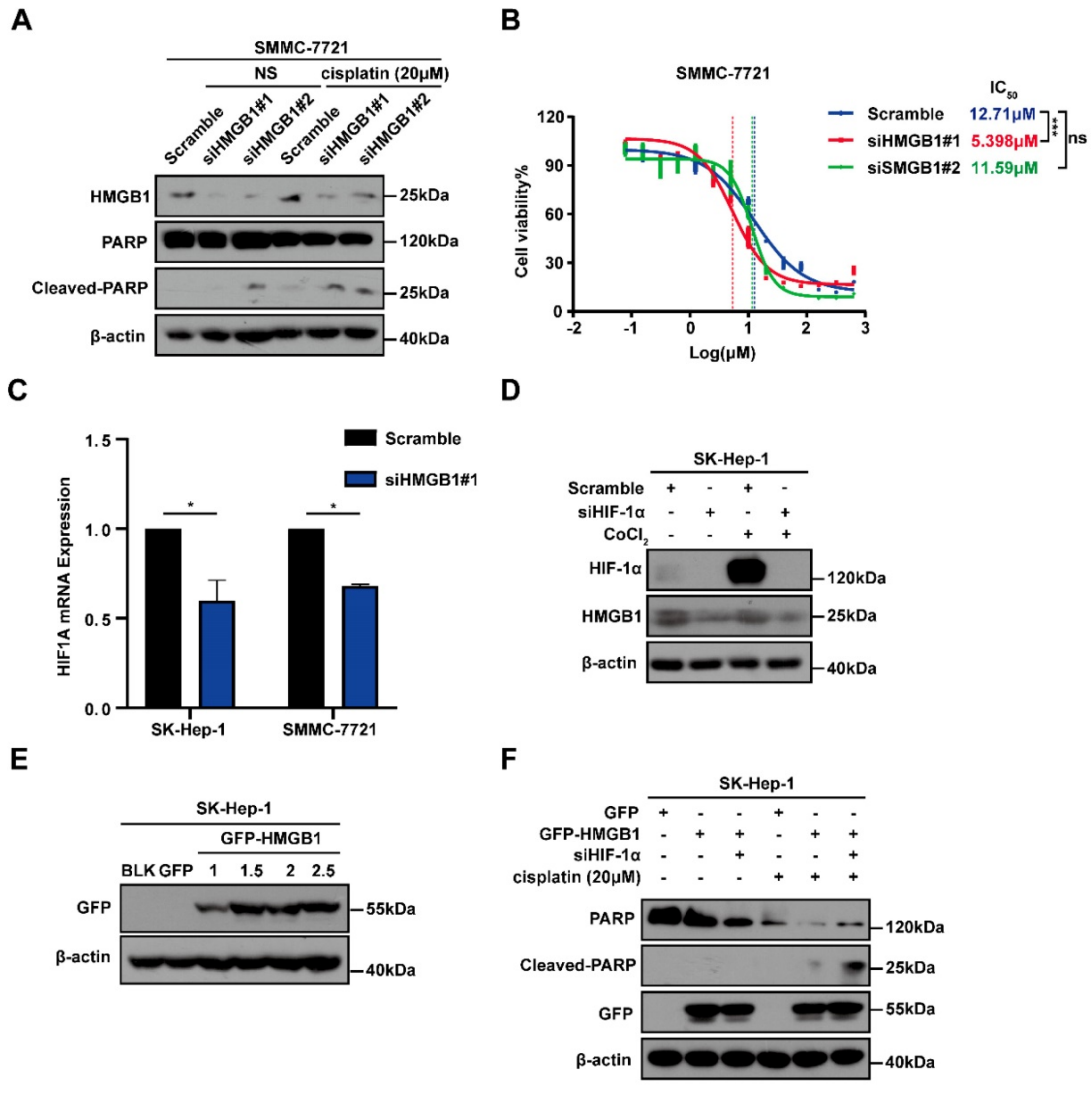
HMGB1 conferred cisplatin resistance to HCC cells via HIF-1α. (A) Lysates from SMMC-7721 cells with or without HMGB1 knockdown after normal saline (NS) or cisplatin treatment (20μM, 24h) were subjected to immunoblotting. (B) Dose response of the cisplatin treatment was assessed by optical density in SMMC-7721 cell lines with or without HMGB1 knockdown. The experiments were performed by triplicate. (C) Total RNAs from SK-Hep-1 and SMMC-7721 cells with or without HMGB1 knockdown were subjected to RT-qPCR. *p* Value was determined by Student's t test (*p < 0.05, ** p < 0.01, *** p < 0.001, ns: not significant). (D) Lysates from SK-Hep-1 cells with or without HIF-1α knockdown and treated with CoCl_2_ (100mM, 24h) were subjected to immunoblotting. (E) Validation of the transfection efficiency of HMGB1 plasmid by immunoblotting. (F) Lysates from SK-Hep-1 cells transfected as indicated and treated with NS or cisplatin (20μM, 24 hours) were subjected to immunoblotting.

To explore the potential involvement of HMGB1 in cisplatin resistance in HCC cells, we first treated SMMC-7721 cells with or without HMGB1 knockdown and subsequently cultured them with normal saline (NS) or cisplatin (20μM) for 24 hours. Using immunoblotting, we detected the apoptosis-related protein cleaved-PARP expression, which showed that HMGB1 silencing promoted cell apoptosis in cisplatin treatment (Fig. 3A). We then examined cisplatin IC_50_ in HCC cells with or without HMGB1 knockdown after culturing them with the indicated cisplatin and using CCK8 assays to verify cell viability. We observed that HMGB1 silencing significantly reduced IC_50_ values as compared with normal controls (Fig. 3B). In the previous experiment, we found that HIF-1α was also upregulated in tumor tissues synchronized with HMGB1 (Fig. 1B). We, therefore, explored the relationship between HMGB1 and HIF-1α. RT-qPCR was used to examine mRNA expression of HIF-1α in HMGB1-silenced HCC cells. The mRNA expression of HIF-1α was downregulated by HMGB1 knockdown in both SK-Hep-1 and SMMC-7721 cells (Fig. 3C). It has been reported that HIF-1α levels were elevated in response to cobalt chloride (CoCl_2_) 16, so we treated cells with CoCl_2_ to mimic overexpression of HIF-1α. Immunoblotting analysis showed that HMGB1 was significantly upregulated under CoCl_2_, and deficiency of HIF-1α suppressed the CoCl_2_-induced increase in HMGB1 expression levels (Fig. 3D). The above results suggested that there is a positive regulatory loop between HMGB1 and HIF-1α. Because HMGB1 induced cisplatin resistance in HCC cells, we speculated that HIF-1α is involved in this process. To validate this hypothesis, we first transfected SK-Hep-1 cells with plasmid to obtain cells with overexpressed HMGB1, verified using immunoblotting (Fig. 3E). We then silenced HIF-1α in overexpressed HMGB1 HCC cells, cultured them with cisplatin (20μM) for 24 hours, and collected the lysates for immunoblotting. The results showed that the protective influence of HMGB1 overexpression on cisplatin-induced cell apoptosis was significantly reversed by HIF-1α deficiency (Fig. 3F). These findings demonstrate that HMGB1 contributed to cisplatin resistance in HCC cells via HIF-1α.

### Positive feedback regulatory loop between HMGB1 and HIF-1α is mediated by NF-κB

**Figure 4:**
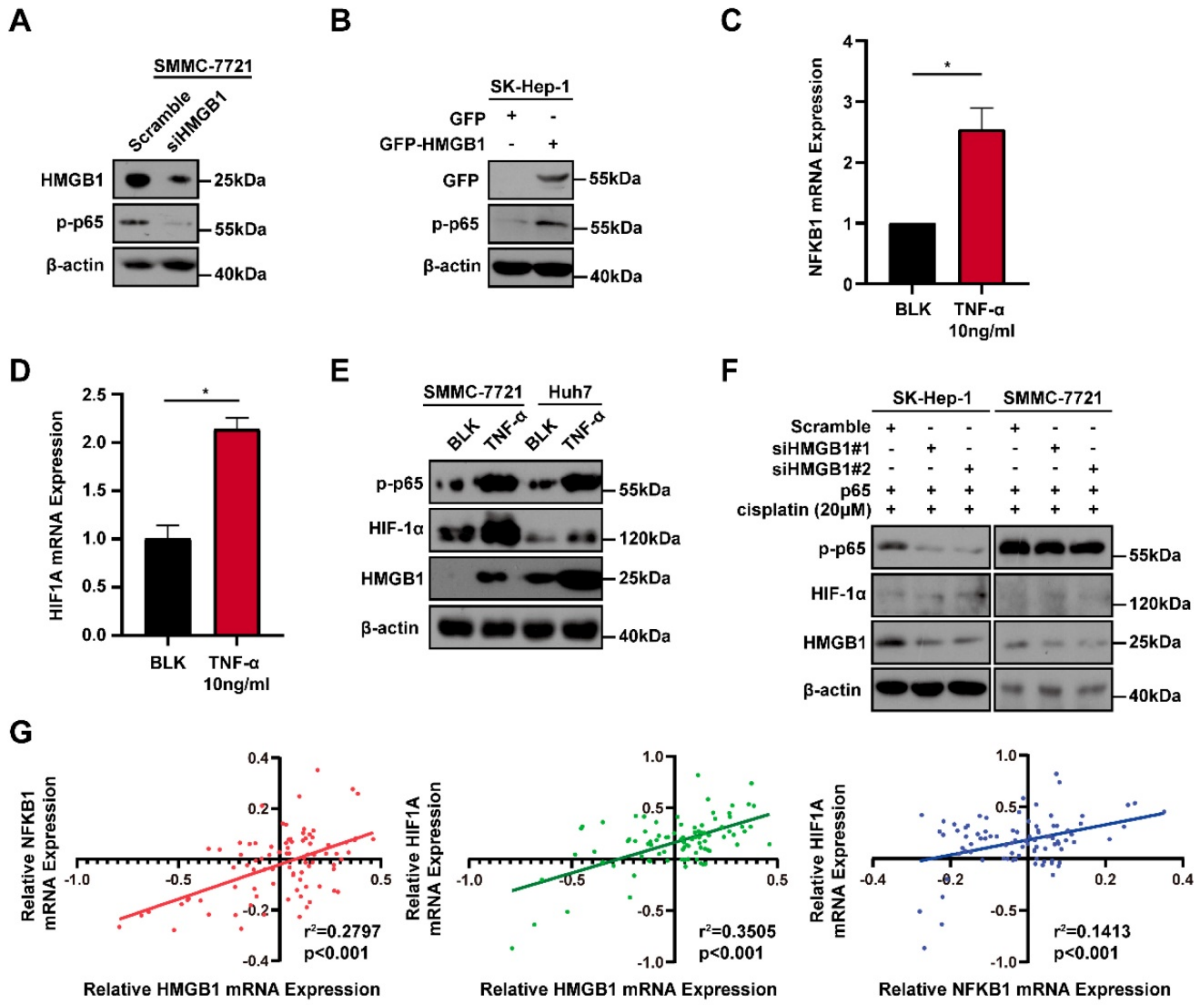
A positive feedback regulatory loop between HMGB1 and HIF-1α is mediated by NF-κB. (A) Lysates from SMMC-7721 cells with or without HMGB1 knockdown were subjected to immunoblotting. p-p65: phosphorylated p65 (B) Lysates from SK-Hep-1 cells transfected with HMGB1 plasmid were subjected to immunoblotting. (C-D) Total RNAs from SMMC-7721 cells treated with TNF-α (10ng/mL, 24h) were subjected to RT-qPCR.* p* Value was determined by Student's t test (*p < 0.05, ** p < 0.01, *** p < 0.001, ns: not significant). (E) Lysates from SMMC-7721 cells and Huh7 cells treated with TNF-α (10ng/mL, 24h) were subjected to immunoblotting. (F) Lysates from SK-Hep-1 and SMMC-7721 cells transfected as indicated and treated with NS or cisplatin (20μM, 24 hours) were subjected to immunoblotting. (G) Correlation analysis among HMGB1, NF-κB and HIF-1α using the GSE364 dataset.

**Figure 5:**
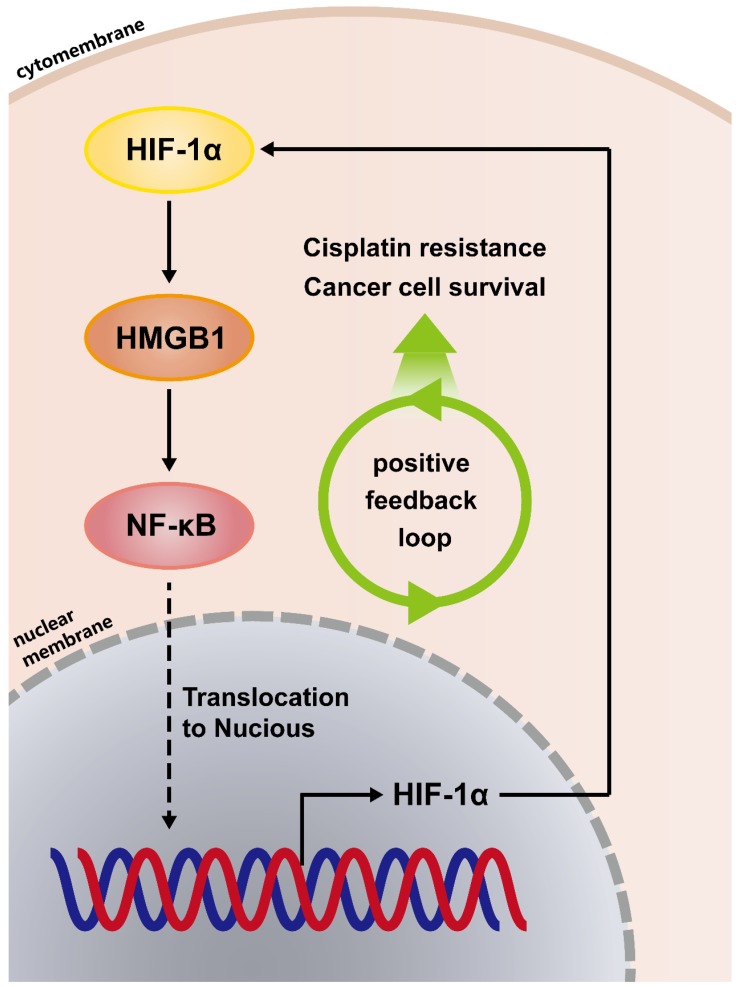
The HMGB1/NF-κB/HIF-1α positive feedback loop makes HMGB1 self-enforcing in HCC cells.

Because the positive regulatory loop between HMGB1 and HIF-1α induced cisplatin resistance, we sought to further explore the mechanism of this loop. The phosphorylated p65 protein level, which is an essential contributor to NF-κB activation was detected using immunoblotting 17. HMGB1 knockdown reduced the phosphorylated p65 level and HMGB1 overexpression increased the phosphorylated p65 level (Figs. 4A and 4B). These results suggest that HMGB1 could activate NF-κB signaling. It has been reported that tumor necrosis factor-α (TNF-α) can activate receptors and trigger potent and persistent activation of NF-қB signaling 18. Therefore, we tested the effect of NF-κB activation on HIF-1α using TNF-α. RT-qPCR showed that the mRNA expressions of NFKB1 and HIF1A were significantly upregulated by TNF-α (10ng/mL) treatment (Figs. 4C and 4D). Furthermore, we detected HIF-1α and HMGB1 protein levels in SMMC-7721 and Huh7 human liver cancer cells after TNF-α (10ng/mL) treatment. Immunoblotting showed that both HIF-1α and HMGB1 were increased in the TNF-α treatment groups (Fig. 4E). We then silenced HMGB1 in p65-overexpressed HCC cells, cultured them with cisplatin (20μM) for 24 hours, and collected the lysates. Immunoblotting showed that the supportive regulation of p65 overexpression on phosphorylated p65 level was reversed when combined with HMGB1 knockdown in both SK-Hep-1 and SMMC-7721 cells. In addition, suppressive regulation of HMGB1 silencing on HIF-1α was rescued in NF-κB-p65 overexpression (Fig. 4F). To confirm their association, we analyzed data from the GSE364 dataset, the linear regression results showed that HMGB1 mRNA expression positively correlated with NF-κB and HIF-1α mRNA levels, NF-κB mRNA expression also positively correlated with HIF-1α mRNA levels (Fig. 4G). Taken together, these results suggest that the HMGB1/NF-κB/HIF-1α positive feedback loop makes HMGB1 self-enforcing in HCC cells (Fig. 5).

## Discussion

Our findings led us to reach several relevant conclusions.

First, we identified that HMGB1 was significantly up-regulated in both cisplatin-treated liver cancer cells and tumor tissues; this, also correlated with poor prognosis in patients with HCC. The results of multivariate analysis results showed that portal vein invasion, alcohol consumption, and HMGB1 expression were significant and independent prognostic factors that could be associated with OS in patients with HCC.

Subsequently, to investigate the potential biological functions of HMGB1 in tumors, we generated HMGB1 knockdown HCC cells. EdU and CCK8 assays showed that reduced HMGB1 expression can inhibit proliferation and promote apoptosis of HCC cells.

Furthermore, we found that HMGB1 was significantly elevated after cisplatin treatment, and HMGB1 deficiency increased the expression of apoptosis-related protein. In addition, our results revealed that the protective influence of HMGB1 overexpression on cisplatin-induced cell apoptosis was significantly reversed by HIF-1α deficiency. These findings indicate that HMGB1 promoted cisplatin resistance in HCC cells via HIF-1α.

Finally, we further explored the mechanism between HIF-1α and HMGB1. HMGB1 could activate NF-κB signaling, and both HIF-1α mRNA and protein levels were increased in the follow-up step. In addition, reciprocally positive connections were found among HMGB1, NF-κB and HIF-1α. As shown in Fig. 5, we found that HMGB1 self-enforces via the HMGB1/NF-κB/HIF-1α positive feedback loop to promote cisplatin resistance in HCC cells.

The role of HMGB1 has been reported by research groups worldwide in the genesis of different cancers, such as lung and breast cancer. Many investigations have suggested that HMGB1 gene polymorphisms are correlated with risk factors of lung cancer development 19. A recent report showed that HMGB1 may be an important factor in the development of chemoresistance in cervical cancer 20. Studies have also reported that chemoresistance of cancer cells is regulated via HMGB1-mediated cell autophagy 21, 22. This study confirmed that HMGB1 promotes cancer and takes part in cisplatin resistance. What is different to the classical HMGB1/RAGE/autophagy pathway, however, is that we verified hepatocellular carcinoma cells resistance to cisplatin treatment by HMGB1 via HMGB1/NF-κB/HIF-1α feedback loop; HMGB1 not only plays the role of cisplatin resistance through this positive feedback loop, but also promotes its high expression in tumor cells.

The NFκB proteins are key regulators of cell proliferation, inhibit apoptosis, promote cell migration and invasion, and stimulate angiogenesis and metastasis 23. NF-κB also regulates the survival and proliferation of tumor cells following cancer chemotherapy 24. When NF-κB was activated, it translocated to the nucleus, where it acted as a nuclear transcription factor 25. It has been shown that NF‐κB is a direct modulator of HIF-1α expression 26. In our study, we verified through RT-qPCR and immunoblotting that TNF-α activated NF‐κB and followed by stimulating HIF-1α.

The nuclear transcription factor, HIF-1α, is a crucial biomarker of the hypoxia tumor microenvironment, which is due to the rapid proliferation of tumor cells 27. Studies have reported that HMGB1 recruited macrophages into HCC tissue via HIF-1α, or HMGB1-regulated HIF-1α to promote the tumor malignancy 28, 29. It can be seen that HMGB1 and HIF-1α act as partners in promoting cancer. Here we reported that the protective influence of HMGB1 overexpression on cisplatin-induced cell apoptosis was significantly reversed by HIF-1α deficiency. Our findings verified that HMGB1 induced cisplatin resistance in HCC cells via HIF-1α, enriched the studies on the role of HMGB1 and HIF-1α in promoting cancer, and it will be interesting to further determine their synergism to induce cisplatin resistance in vivo.

It has been demonstrated that NF-κB and HIF-1α play a role in the development of many cancers, such as LMP1-induced HIF-1α transcription via ERK1/2/NF-κB pathway in nasopharyngeal carcinoma cells to promote angiogenesis^30^; metformin-sensitized response of oral squamous cell carcinoma to cisplatin treatment through inhibition of the NF-κB/HIF-1α signal axis^31^, and our study finding that show HMGB1-involvement in the NF-κB/HIF-1α pathway, which caused cisplatin resistance in HCC cells. It has been shown that extracellular HMGB1 induces angiogenesis and promotes rheumatoid arthritis via NF-κB/HIF-1α activation, and cilostazol inhibits NF-κB-mediated transcription leading to the inhibition of synovial angiogenesis 32. In our study, we provide new evidence of a cytoplasmic HMGB1 regulation mechanism whereby a HMGB1/NF-κB/HIF-1α feedback loop regulates HMGB1 expression and function in HCC cells, thus suggesting that suppressing the link in this loop may stop the vicious circle and achieve therapeutic effects.

In summary, for the first time, we confirmed a reciprocal regulatory interaction loop between HMGB1, NF-κB, and HIF-1α, which implements HMGB1 self-enforcing, leading to cisplatin resistance in HCC. The inhibition of this positive regulatory loop could be a valuable pharmaceutical target to disrupt HMGB1 self-enforcing and reversing cisplatin resistance in clinical antitumor therapeutics 33.

## Figures and Tables

**Table 1:** The associations of HMGB1 expression with clinicopathological characteristics in ICGC-LIRI-JP (n=203) cohort

Feature	Low expression of HMGB1 (n=150)	High expression of HMGB1 (n=53)
Age(years)		
≥50	139	47
<50	11	6
Gender		
Male	109	44
Female	41	9
Virus		
HBV	35	18
HCV	88	29
HBV and HCV	2	2
Negative	25	4
TNM stage		
I+II	101	28
III+IV	49	25
Portal vein invasion		
Positive	35	14
Negative	115	39
Vein invasion		
Positive	25	12
Negative	125	41
Bile duct invasion		
Positive	9	4
Negative	141	49
Fibrosis		
Yes	142	52
No	8	1
Alcohol		
Yes	58	32
No	84	20
NA	8	1
Smoking		
Yes	78	36
No	65	16
NA	7	1

Abbreviations: NA, not available.

**Table 2:** Univariate and multivariate analyses of OS in ICGC-LIRI-JP (n=203) cohort by Cox regression analysis

Variables	Univariate analysis				Multivariate analysis		
	HR	CI (95%)	*P* value		HR	CI (95%)	*P* value
Age (years)	0.922	0.325-2.616	0.878				
Gender	0.536	0.265-1.083	0.082				
Virus (HBV or/and HCV)	1.654	0.506-5.408	0.406				
TNM stage	2.830	1.446-5.540	0.002*				
Portal vein invasion	3.135	1.595-6.160	0.001*		2.395	1.109-5.169	0.026*
Vein invasion Bile duct invasion	2.394 0.531	1.172-4.891 0.072-3.890	0.017* 0.533				
Fibrosis	1.158	0.276-4.853	0.841				
Alcohol	0.476	0.242-0.936	0.031*		0.390	0.193-0.786	0.009*
Smoking	0.758	0.391-1.471	0.413				
HMGB1	2.162	1.098-4.255	0.026*		2.192	1.102-4.363	0.025*

Abbreviations: HBV, hepatitis B virus; HCV, hepatitis C virus; CI, confidence interval; HR, hazard radio; *The values had statistically significant differences.
